# P-2076. Understanding the Impact of Patient Factors in Infectious Diseases Referral Completion

**DOI:** 10.1093/ofid/ofaf695.2240

**Published:** 2026-01-11

**Authors:** Karina Garcia, Rose Bethanica Gelin, Rayven Frierson, Djenabou Sow, Elinel Gonzalez-Baez, Jennifer A Johnson, Sophia Koo

**Affiliations:** Brigham and Women's Hospital, San Diego, CA; Student Success Job Program, Brockton, Massachusetts; Brigham & Women's Hospital, Boston, Massachusetts; Brigham & Women's Hospital, Boston, Massachusetts; Brigham & Women's Hospital, Boston, Massachusetts; Brigham And Women's Hospital / Dana-Farber Cancer Institute / Harvard Medical School, Boston, Massachusetts; Brigham and Women's Hospital, Dana-Farber Cancer Institute, Boston, MA

## Abstract

**Background:**

Many patients face barriers to accessing optimal care of their infections, such as HIV/AIDS, tuberculosis, and COVID-19, where prompt diagnosis and treatment are crucial. We investigated the association between demographic characteristics and non-completion of referrals to our ID clinic for consultation, requested by each patient’s clinical care team.

**Table 1:**
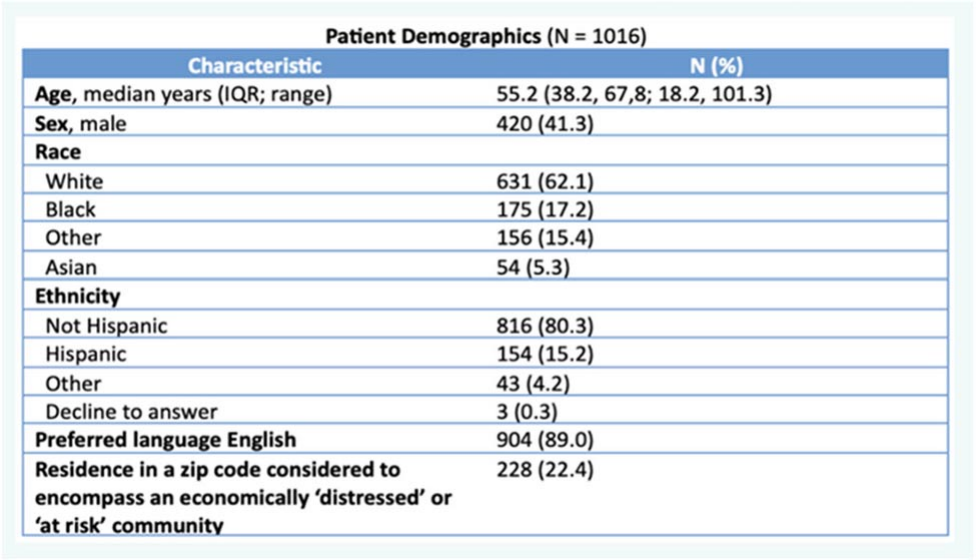
Patient characteristics.

**Figure 1:**
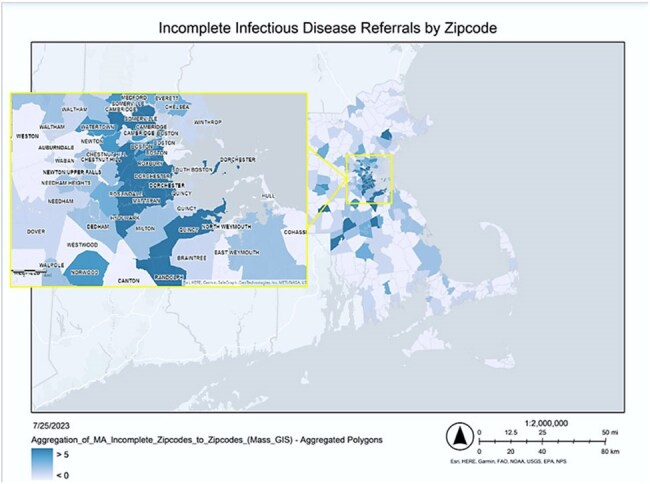
Aggregation maps of ID clinic referral non-completion rates in Massachusetts zip codes. The color scale ranges from light blue, representing low rates of incomplete referrals, to dark blue, indicating high rates of incomplete referrals (ArcGIS, 2024).

**Methods:**

We conducted a retrospective study assessing demographic and clinical parameters in all patients who had electronic referrals placed by a medical provider for ambulatory consultation at an ID clinic associated with a large tertiary academic medical center between May 2021- May 2023. We analyzed the association between these parameters and non-completion of clinic referrals using univariable and multivariable logistic regression models and used ArcGIS for spatial mapping to assess the association between patient zip codes and non-completion of referrals.

**Table 2:**
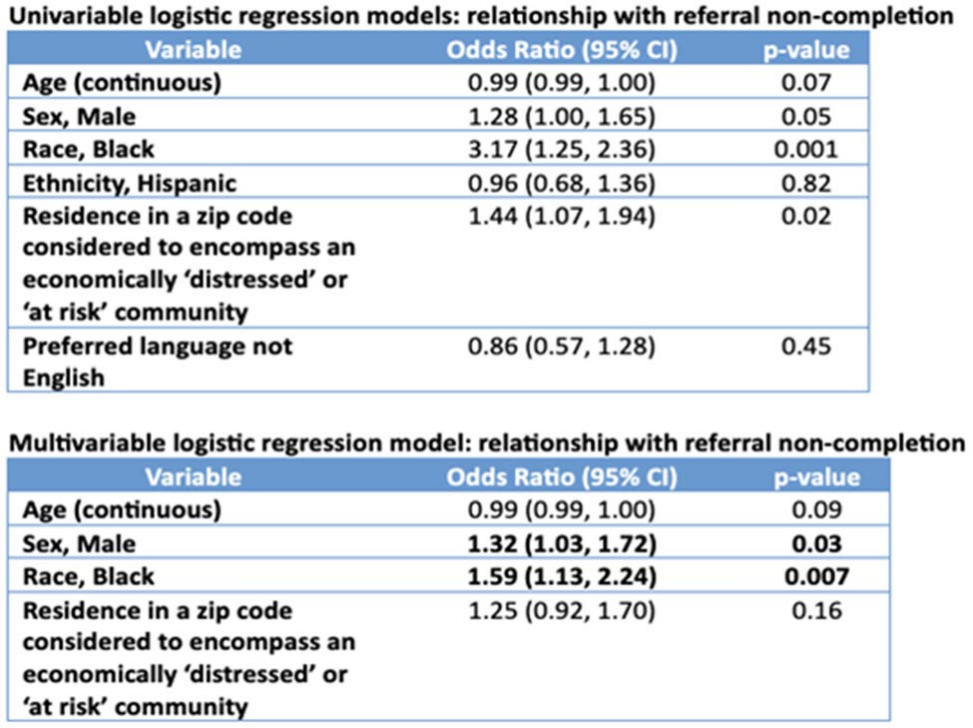
Logistic regression models examining the relationship of demographic and socioeconomic indicators with ID clinic referral non-completion.

**Results:**

Of 1016 patients referred electronically for ambulatory ID consultation over this period (Table 1), 414 (40.7%) referrals were considered non-complete (the patient was never evaluated in the ID clinic despite a clinical provider requesting an evaluation). In univariable models, odds of non-completion were 3.17-fold higher among Black compared to non-Black individuals, and 1.44-fold higher in residents of zip codes considered ‘at-risk’ or ‘distressed’ (Table 2). In the multivariable model, odds of non-completion were 1.32-fold higher in males and 1.59-fold higher in Black individuals, adjusting for age and residence in an economically disadvantaged zip code.

**Conclusion:**

The odds of non-completion of referrals to our ID clinic were significantly higher in males and Black individuals, adjusting for other factors. We identified these subgroups as important outreach targets for collaborative efforts to improve referral completion and health equity in our clinic.

**Disclosures:**

Sophia Koo, MD, SM, Ansun BioPharma: Grant/Research Support|Generate Biomedicines: Advisor/Consultant|GlaxoSmithKline: Grant/Research Support|Locus Biosciences: Grant/Research Support|Merck Sharp & Dohme: Grant/Research Support|Scynexis, Inc: Grant/Research Support|Vertex Pharmaceuticals: Advisor/Consultant

